# Experimental selection of long-term intracellular mycobacteria

**DOI:** 10.1111/cmi.12303

**Published:** 2014-06-02

**Authors:** Cristina L Vázquez, Thomas R Lerner, Bahram Kasmapour, Gang Pei, Achim Gronow, Maria V Bianco, Federico C Blanco, Christopher K E Bleck, Robert Geffers, Fabiana Bigi, Wolf-Rainer Abraham, Maximiliano G Gutierrez

**Affiliations:** 1 Research Group Phagosome Biology, Helmholtz Centre for Infection ResearchInhoffenstrasse 7, 38124, Braunschweig, Germany; 2 Division of Mycobacterial Research, Medical Research Council, National Institute for Medical ResearchLondon, NW7 1AA, UK; 3 Instituto de Biotecnología, CICVyA-INTAN. Repetto and Los Reseros, 1686, Hurlingham, Argentina; 4 Centre for Cellular Imaging and NanoAnalytics, Structural Biology and Biophysics, Biozentrum, Mattenstrasse 26 University of BaselCH-4058, Basel, Switzerland; 5 Research Group Genome Analytics, Helmholtz Centre for Infection ResearchInhoffenstrasse 7, 38124, Braunschweig, Germany; 6 Research Group Chemical Microbiology, Helmholtz Centre for Infection ResearchInhoffenstrasse 7, 38124, Braunschweig, Germany

## Abstract

Some intracellular bacteria are known to cause long-term infections that last decades without compromising the viability of the host. Although of critical importance, the adaptations that intracellular bacteria undergo during this long process of residence in a host cell environment remain obscure. Here, we report a novel experimental approach to study the adaptations of mycobacteria imposed by a long-term intracellular lifestyle. Selected *Mycobacterium bovis* BCG through continuous culture in macrophages underwent an adaptation process leading to impaired phenolic glycolipids (PGL) synthesis, improved usage of glucose as a carbon source and accumulation of neutral lipids. These changes correlated with increased survival of mycobacteria in macrophages and mice during re-infection and also with the specific expression of stress- and survival-related genes. Our findings identify bacterial traits implicated in the establishment of long-term cellular infections and represent a tool for understanding the physiological states and the environment that bacteria face living in fluctuating intracellular environments.

## Introduction

A number of intracellular bacteria are able to induce life-long infections in their hosts (Stewart *et al*., 2003; Monack *et al*., 2004). An important consequence of an intracellular habitat is that bacteria are exposed to a fluctuating host cell environment for long periods of time. Intracellular bacteria can also face extremely different conditions during switching between temporary extra- and intracellular states (Casadevall, 2008). Although intracellular bacteria can be localized in the cytoplasm, commonly the environment they face is a modified vacuolar compartment (Flannagan *et al*., 2009). Therefore bacteria habiting host cells must somehow adapt to this long-term permanent lifestyle (Honer zu Bentrup and Russell, 2001; Casadevall, 2008) by re-wiring metabolic networks necessary to survive for years within hosts (Honer zu Bentrup and Russell, 2001). This important phenomenon is poorly understood and the phenotypic and genetic changes imprinted in long-term intracellular bacteria are ill defined (Casadevall, 2008).

It has been proposed that intracellular pathogenesis is a part of a broad range of intracellular adaptation strategies in a constant co-evolution of both hosts and pathogens (Casadevall, 2008). Although broadly used to study cellular adaptations of viruses (An *et al*., 2006; Wang and Damania, 2008), experimentally accessible cellular systems to study the adaptations of intracellular bacteria are not well developed. Bacterial changes have been monitored *in vitro* in infected cells for periods of weeks to 1 year (Ensminger *et al*., 2012; Rohde *et al*., 2012) but systems to evaluate longer processes of selection/adaptation are not well established.

In order to investigate the adaptations of mycobacteria to an intracellular environment, we developed a cellular system that allowed *Mycobacterium bovis* BCG to continuously reside in macrophages for years at very low numbers. BCG has long been used as a vaccine against its close relative *Mycobacterium tuberculosis*. Whereas *M. tuberculosis* is a biosafety level 3 organism, BCG is generally not considered a pathogen. However, both *M. tuberculosis* and BCG share important characteristics such as the ability to arrest the phagolysosomal fusion and persist under certain conditions in host macrophages (Fritz *et al*., 2002; Leversen *et al*., 2012).

Using this approach, we provide in this report insights into important principles of this long process of intracellular adaptation. Long-term infection of macrophages in culture was maintained primarily through processes of host cell death followed by efferocytosis and infected host cell division associated with controlled bacterial cell division. We found that this long-term infection selected mycobacteria that were preferentially localized in a harsh phagolysosomal compartment with autophagic features. Isolated long-term selected intracellular bacteria have an improved ability to metabolize glucose and accumulate neutral lipids *in vitro*. Moreover, selected bacteria did not produce PGL as a result of a frameshift insertion polymorphism in the *pks1* gene. Strikingly, upon re-infection of host cells and mice, mycobacteria with the here-described adaptations were more likely to survive not only in macrophages but are also able to reside longer in mice. Finally, a gene expression analysis identified stress-induced targets that are selectively regulated during re-infection and can potentially help the process of adaptation.

## Results

### Long-term experimental selection of mycobacteria in cultured macrophages

**Figure 1:**
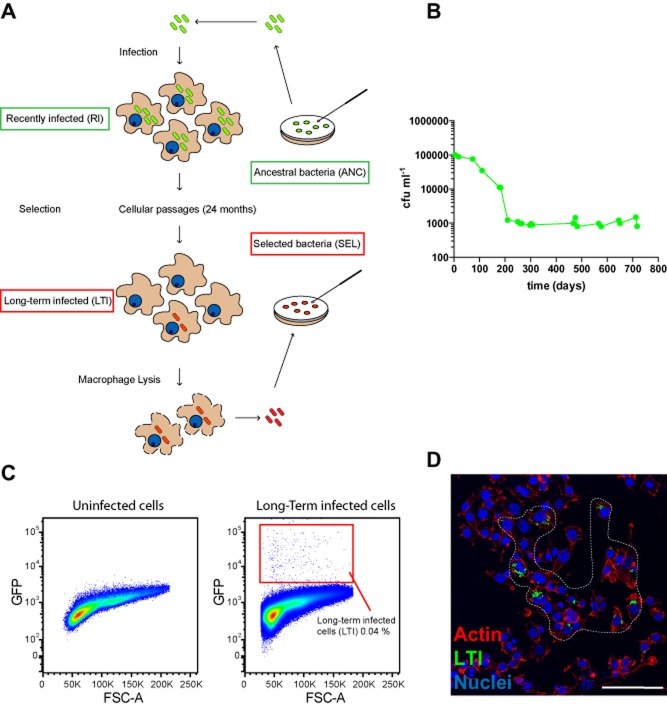
Long-term experimental selection of mycobacteria in cultured macrophages.A. Strategy followed to select mycobacteria in RAW 264.7 macrophages for extended periods of time.B. Colony-forming units (cfu) of BCG-GFP at the selected time points during the first 2 years of the process of mycobacterial selection in macrophages through successive passages.C. Representative flow cytometry plot showing a culture of uninfected cells (left panel), and the percentage of infected cells in long-term infected macrophage culture (right panel). Two-year-old infected macrophages were used for these experiments.D. Representative image showing a typical group of long-term infected macrophages in culture. Infected macrophages were fixed and stained for actin with Rhodamine-phalloidin and for nuclei with Hoechst 33258. Scale bar: 50 μm.

To study the impact of a long-term intracellular habitat, we generated a culture of RAW 264.7 macrophages continuously infected with *M. bovis* BCG constitutively expressing GFP (BCG-GFP). This system represents a relevant and tractable system to study bacteria-macrophage interactions (Gutierrez *et al*., 2004). Macrophages were infected with BCG-GFP at a multiplicity of infection (MOI) of 10. The starting cultures of bacteria used for infection were preserved in frozen aliquots representing the ‘ancestral’ bacteria (ANC, Fig. 1A). After infection, macrophages were washed, split and continuously re-plated when confluent during a period of at least 2 years. We then defined macrophages infected with ANC for 24 h as ‘recently infected’ (RI, Fig. 1A); macrophages infected for at least 2 years as ‘long-term infected’ (LTI, Fig. 1A) and bacteria recovered from LTI as ‘selected’ mycobacteria (SEL, Fig. 1A). We observed that viable mycobacteria remained associated with these macrophage cultures (Fig. 1B). Mycobacteria stayed mostly in an intracellular environment since gentamicin treatment did not reduce the numbers of viable bacteria from infected cells (Fig. S1A–B). In addition, supernatants of the long-term infected culture were negative for colonies (Fig. S1C–D). Bacteria were found to grow slowly as measured by the intensity of fluorescence associated to single macrophages by live cell imaging (Fig. S1E). The number of bacteria recovered from infected macrophage cultures dropped dramatically between 3 and 7 months of infection (Fig. 1B). Subsequently, the number of infected cells and viable bacteria remained constant but very low at a stable plateau until 12 months and onwards. Strikingly, after 1 year, approximately 0.04% of the total cells were infected as determined by flow cytometry (Fig. 1C). By microscopy, this low number of infected macrophages was primarily found in distinct groups of 5–12 cells (Fig. 1D). Altogether, our data indicated that in this system both macrophages and mycobacteria contributed to the dynamic process of establishment and stabilization of the long-term infection.

### Host cell mechanisms contributing to the maintenance of a long-term infection

**Figure 2:**
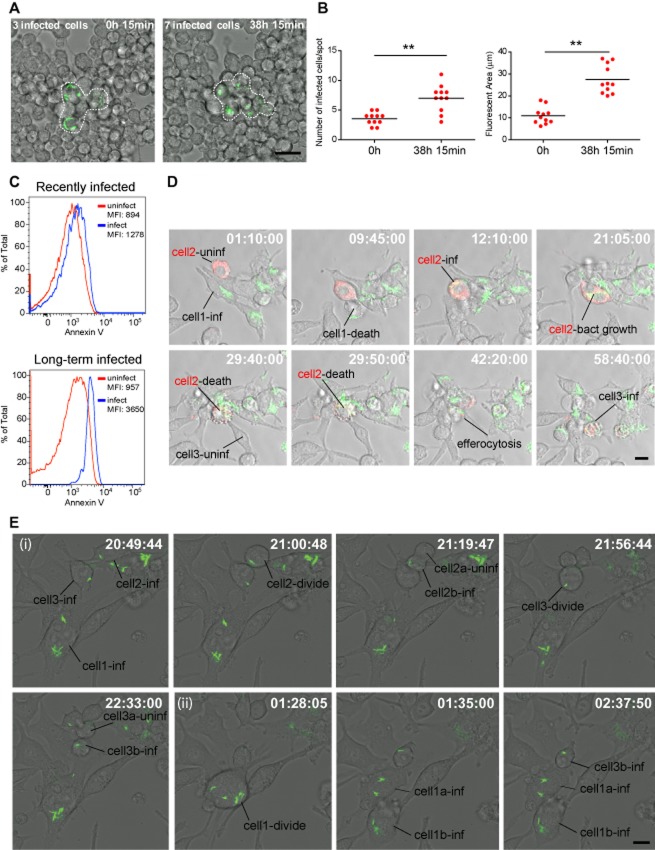
Host cell mechanisms contributing to the maintenance of a long-term infection.A. Initial and final frames of Movie S1 showing the expansion of a group of three infected cells into seven infected cells in a period of 38.15 h. Scale bar: 10 μm.B. Quantitative analysis of the changes in the number of infected cells and fluorescent area occupied by BCG-GFP, the data are from 11 movies. (**) *P* ≤ 0.01 from two-tailed Student's *t*-test.C. Analysis by flow cytometry of LTI and RI macrophages showing Annexin V signal intensity distribution. In order to determine cell death, cells were double stained with Annexin V and 7-AAD and gated based on 7-AAD positive signal and GFP-positive signal (infected cells, blue line) or GFP-negative (uninfected cells, red line). Results are represented as % of total in the *y*-axis versus intensity of Annexin V signal in *x*-axis. The median fluorescence intensity (MFI) values for each gated population are shown in the histograms. Data are representatives of three independent experiments.D. Time-lapse of Movie S2 showing cell death and efferocytosis as a mechanism for propagation of the infection in the culture of long-term infected macrophages. Scale bar: 10 μm.E. Time-lapse of Movie S3 showing different events of cell division in long-term infected macrophages and distribution of selected bacteria. (i) Infected cell divides and bacteria are segregated into just one daughter cell. (ii) Infected cell divides and bacteria are segregated into both daughter cells. Scale bar: 10 μm.

We then investigated how this long-term equilibrium between macrophages and mycobacteria is maintained in the distinct, small groups of infected cells. For that, we performed a dynamic analysis by live cell imaging over a period of 5 days on LTI macrophages (Fig. 2A). Twenty-four hours after seeding (time point 0 h), the number of infected cells in the infected cell groups was on average 4, and then after 38 h 15 min, they significantly increased to an average of seven infected cells, correlating with a significant increase in the total fluorescent area (Fig. 2B, Movie S1). These results indicate that despite the different passages of the long-term culture, the number of infected cells is maintained through the time. By adding uninfected RAW 264.7 macrophages stably expressing an Endoplasmic reticulum (ER)-mCherry marker to the flask containing LTI macrophages (see *Experimental procedures*), we identified host cell death followed by efferocytosis via neighbouring cells as a mechanism by which the number of infected cells temporarily increased and sustained the LTI cells (Fig. 2D). In this process, single heavily infected macrophages went through cell death without detachment and subsequently other macrophages efferocytosed the bacteria present in the dying cell. As a result, from single infected macrophages, up to five to six infected macrophages were generated (Fig. 2D, Movie S2). In agreement with this interpretation, we observed that LTI macrophages were both positive for the membrane integrity and apoptotic markers 7-aminoactinomycin D (7-AAD) and Annexin V respectively, when compared with RI macrophages (Fig. 2C). Additionally, when LTI cells divided, the bacteria-containing phagosomes were also segregated into the cytoplasm of the daughter cells (Fig. 2E, Movie S3). Thus, mycobacteria were also transferred during the replication of host macrophages. However, often when cells divided, the phagosomes were all distributed to one single cell (Fig. 2E, Movie S3). As a result, the expansion process of infected cells was rather heterogeneous since not all the groups of infected cells expanded. Therefore, both cell death followed by efferocytosis and cell division contributed to maintain the number of infected cells in the long term.

### Selected bacteria reside in LC3-positive phagolysosomes

**Figure 3:**
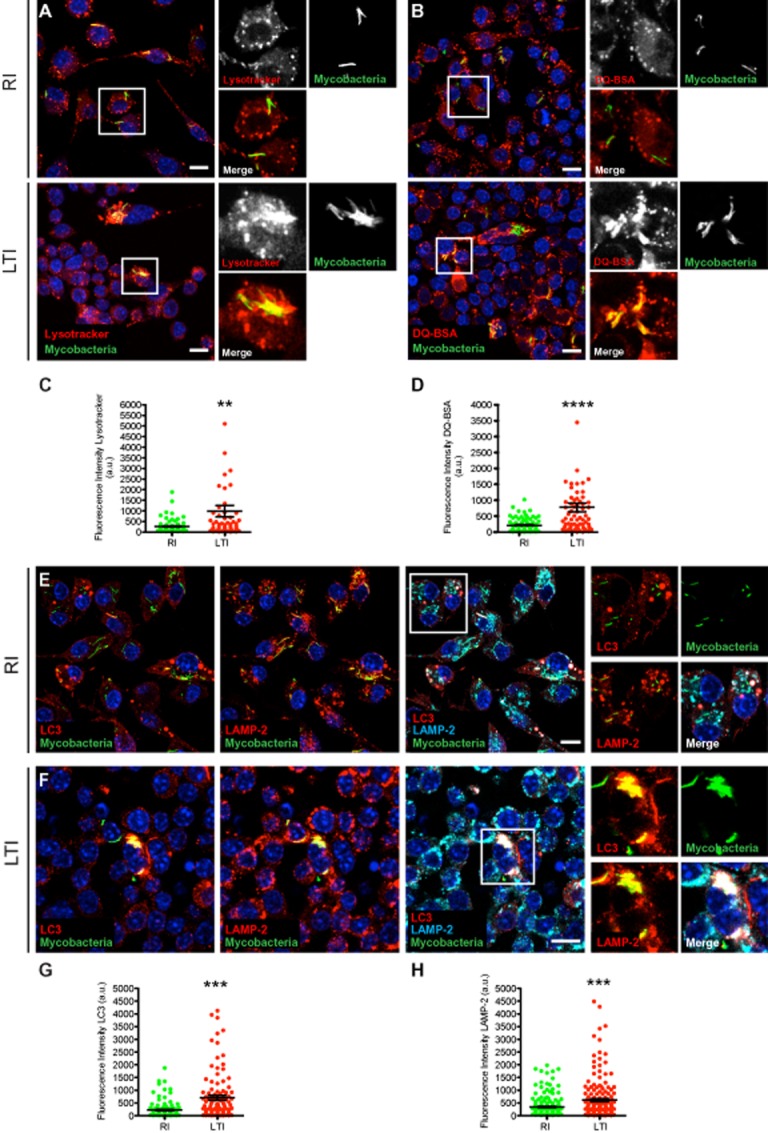
Selected bacteria reside in LC3-positive phagolysosomes.A and B. Long-term infected (LTI) macrophages for at least 2 years and recently infected (RI) with BCG-GFP ancestral strain for 24 h were stained with Lysotracker (A, left panel) or DQ-BSA (B, right panel).C and D. Quantitative analysis of the fluorescence intensity of Lysotracker (C) and DQ-BSA (D) associated with phagosomes in LTI macrophages compared with RI cells. Around 80 phagosomes were quantified in Lysotracker or DQ-BSA stained macrophages from three independent experiments.E and F. Recently infected macrophages (RI) during 24 h (E) and LTI (F) macrophages (2 years old) were subjected to immunofluorescence and endogenous LC3 and LAMP-2 were detected using specific antibodies. Insets depict colocalization with single markers.G and H. Quantitative analysis of the fluorescence intensity of LC3 (G) and LAMP-2 (H) associated with phagosomes of RI and LTI macrophages. Around 130 phagosomes were quantified for LC3 detection and 190 phagosomes for LAMP-2 detection from three independent experiments.Data represent the mean ± S.E.M., (**) *P* ≤ 0.01, (***) *P* ≤ 0.001 and (****) *P* ≤ 0.0001 from two-tailed Student's *t*-test. Nuclei were stained with Hoechst 33258. Scale bars: 10 μm.

In RI macrophages, approximately 30% of mycobacteria localized in early phagosomes as previously reported (Sturgill-Koszycki *et al*., 1994; Via *et al*., 1998; Gutierrez *et al*., 2004) (data not shown). A very low association of Lysotracker with mycobacterial phagosomes was observed in RI macrophages (Fig. 3A and C). In contrast, in LTI macrophages, association of Lysotracker with mycobacterial phagosomes was significantly higher (Fig. 3A and C). Moreover, whereas mycobacterial phagosomes in RI macrophages acquired low levels of Self-quenched BODIPY dye conjugated to bovine serum, (DQ-BSA) from degradative compartments, phagosomes in LTI macrophages acquired significantly higher levels of DQ-BSA (Fig. 3B and D). Mycobacterial phagosomes in LTI were positives for the autophagic protein LC3 as well as the late endocytic marker LAMP-2 (Fig. 3E–H). At the ultrastructural level, mycobacteria within the LTI macrophages were located in membrane-enclosed compartments resembling late endocytic and/or autophagocytic organelles (Fig. S2A–B). Thus, in LTI cells, bacteria mostly reside in hydrolytic/autophagic compartments, suggesting that after this process of selection, mycobacteria adapted to this hostile environment. We therefore hypothesized that this long period of residence in an intracellular state, may have selected bacteria able to remain within macrophages and adapt to the phagosomal environment.

### Phenotypic and genetic changes of selected mycobacteria

**Figure 4:**
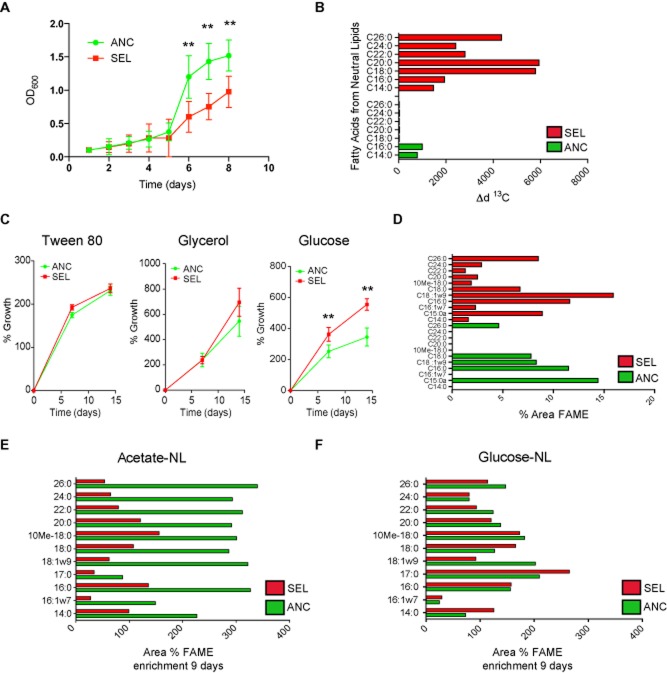
Phenotypic and genetic changes of selected mycobacteria.A. *In vitro* growth of both ancestral and selected bacteria in complete 7H9 medium. Curves represent the mean ± S.E.M. of four independent experiments, (**) *P* ≤ 0.01 from two-tailed Student's *t*-test.B. Isotopic labelling of fatty acids according to the carbon length associated to neutral lipids after 1 day of incorporation of ^13^C-acetate in complete 7H9 medium. The plot shows the incorporation of ^13^C-acetate into neutral lipids in ANC and SEL bacteria (*x*-axis).C. *In vitro* growth of ancestral and selected bacteria in minimal medium (Sauton) containing Tween 80, glycerol or glucose as a sole carbon source during 7 and 14 days. Data represent the mean ± S.E.M. of at least three independent experiments, (**) *P* ≤ 0.01 from two-tailed Student's *t*-test.D. Percentage of the fatty acid methyl esters (FAME) area associated to neutral lipids and corresponding to the indicated carbon length for ancestral and selected strains growing in minimal Sauton medium after 1 day of incorporation of ^13^C-glucose as sole carbon source.E. Enrichment into fatty acids associated to neutral lipids after 9 days of growth in ancestral and selected strains growing in Sauton medium with ^13^C-acetate as sole carbon source.F. Enrichment into fatty acids associated to neutral lipids after 9 days of growth in ancestral and selected strains growing in Sauton medium with ^13^C-glucose as sole carbon source.

We next focused on understanding the mechanisms that allowed these selected bacteria to remain viable in this dynamic environment of macrophages in long-term culture. SEL mycobacteria grew significantly slower in complete Middlebrook 7H9 medium compared to the ancestral bacteria (Fig. 4A). At the ultrastructural level, we found by electron microscopy that SEL mycobacteria within LTI macrophages contained high amounts of intracellular lipid droplets (Fig. S2C–D) positive for the neutral lipid stain Nile red (Fig. S2E–F). To further understand this change in neutral lipid accumulation, the flow of substrates was traced *in vitro* by comparing isolated ancestral and selected bacteria. By using gas chromatography coupled to isotopic ratio-mass spectrometry (GC-IRMS), we found that selected bacteria were able to incorporate higher amounts of ^13^C-acetate into neutral lipids in complete Middlebrook 7H9 medium *in vitro* at a higher rate (Fig. 4B). The effect was specific since no significant changes were observed in the synthesis of glycolipids and phospholipids (Fig. S3). Thus, SEL bacteria synthesized and accumulated neutral lipids faster than the ANC bacteria. A switch in carbon-source usage is postulated for bacteria that changed from an extra- to an intracellular niche (Boshoff and Barry, 2005; Eisenreich *et al*., 2010). We found that the growth of SEL bacteria in minimal Sauton medium was significantly faster than the ANC bacteria in glucose but not in glycerol or Tween 80 (fatty acids donor) as a sole carbon-source, indicating a switch in carbon source usage to glycolytic substrates (Fig. 4C). By using GC-IRMS, in minimal medium containing glucose as a carbon source, the incorporation of ^13^C-glucose after 1 day in selected bacteria was associated with specific long carbon chain fatty acids of neutral lipids (Fig. 4D). In contrast, when we tested the enrichment into fatty acids associated to neutral lipids using ^13^C-acetate as sole carbon source, we observed lower levels of incorporation in the selected bacteria compared to the ancestral bacteria after 9 days (Fig. 4E). However, the enrichment of ^13^C-glucose incorporation in SEL bacteria was similar or higher compared to the ANC bacteria (Fig. 4F). Altogether, our data showed that SEL mycobacteria have an altered ability to use carbon sources with a preference for glucose and storage of neutral lipids in droplets.

Next, we re-sequenced the genome of both ANC and SEL mycobacteria. We mapped both genomes against the original strain *M. bovis* BCG str. Pasteur 1173P2 (Fig. S4A). Genome coverage averaged 96% with an average read depth of 100 across the genomes. Our single-nucleotide polymorphism (SNPs) analysis revealed 18 single-nucleotide differences (coverage > 20) in the two strains compared to the reference Pasteur 1173P2. However, we found no significant differences between them (see pair-wise comparison, Table S1). We concluded these SNPs represent the mutational history of the Pasteur 1173P2 strain we had in our laboratory. On the other hand, the small nucleotide deletion-insertion (DIP) analysis (coverage > 20) revealed three DIPs between the ANC mycobacteria and Pasteur 1173P2 and four DIPs between the SEL mycobacteria and Pasteur 1173P2 (Table S2). Only one single DIP was found to be different between the ancestral and selected bacteria. This unique insertion of a guanine nucleotide generated a frameshift in the *pks1* gene of the selected strain confirmed by Sanger sequencing in at least 10 different colonies of selected bacteria isolated from long-term infected macrophages (data not shown). We confirmed by thin-layer chromatography the lack of PGL production in the selected mycobacteria compared to the ancestral bacteria (Fig. S4B).

### Adaptation of selected mycobacteria during re-infection

**Figure 5:**
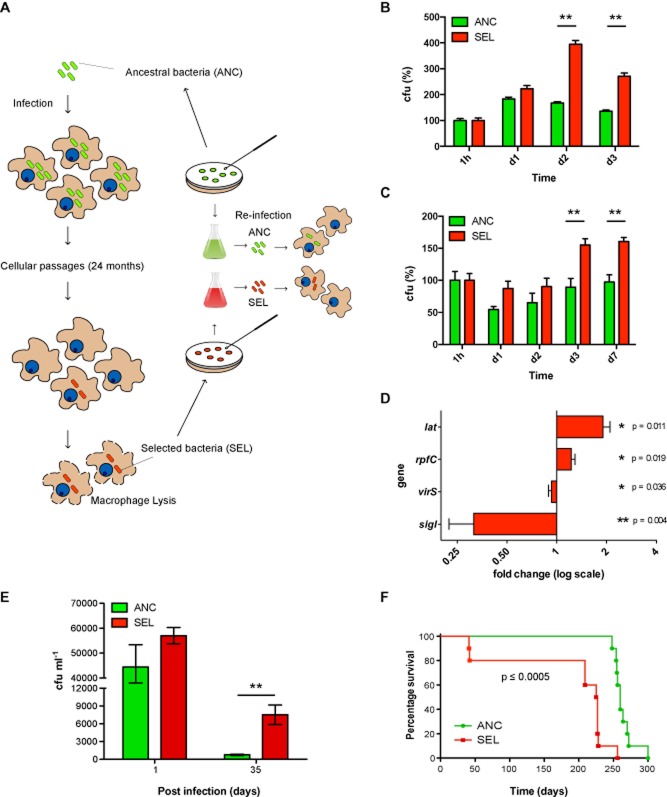
Adaptation of selected mycobacteria during re-infection.A. Diagram showing the strategy followed in the re-infection experiments.B. Equal amounts of ancestral (ANC) and selected (SEL) bacteria were used to re-infect RAW 264.7 macrophages as indicated in experimental procedures. After 1, 2 and 3 days of infection, the number of cfu were calculated.C. Ancestral and selected bacteria were used to re-infect primary bone marrow macrophages (BMMs) as indicated in *Experimental procedures*. After 1, 2, 3 and 7 days of infection, the number of colonies was counted and cfu were calculated.Data represent the mean ± S.E.M., (**) *P* ≤ 0.01 from two-tailed Student's *t*-test from three independent experiments.D. Quantitative PCR (qPCR) analysis of the expression of selected *M. bovis* BCG genes. Data represent the mean ± SD from four independent experiments. Student's *t*-tests were performed to verify the differences in the fold change in gene expression.E. Lung bacillary loads in BALB/c mice after intratracheal inoculation (1.25 × 10^5^ cfu) of selected (red bars) or ancestral bacteria (green bars). The data represent the mean number of cfu ± SD in five mice of one representative experiment out of two independent experiments, (**) *P* ≤ 0.01 from two-tailed Student's *t*-test.F. Survival of Nude mice after intratracheal inoculation with (1.25 × 10^4^ cfu) of selected (red bars) or ancestral (green bars) bacteria. Statistical analysis for survival curves was performed using Mantel-Cox test *P* ≤ 0.0005.

Our findings raised the question of how these selected metabolic and genotypic changes may affect bacterial adaptation during re-infection. To address this, we re-infected macrophages with the ANC and SEL bacteria in order to evaluate their survival (Fig. 5A). We found a significant increase in the number of intracellular mycobacteria in RAW 264.7 macrophages infected with SEL compared to ANC bacteria (Fig. 5B) although no differences were observed in internalization rates between ancestral and selected bacteria (data not shown). Consistently, the numbers of selected mycobacteria were higher than the ancestral mycobacteria in primary bone marrow macrophages (BMMs) after 7 days of infection (Fig. 5C). In order to confirm that the *pks1* gene mutation is involved in the mycobacterial adaptation to host cells, we deleted the *pks1* gene from the ANC bacteria. Unfortunately, several attempts to complement the mutant and the selected BCG strains with an intact copy of *pks1* gene were unsuccessful possibly because the changes in cell wall composition (data not shown). We generated two Δ*pks1* ANC strains, BCG ANC Δ*pks1*-1 and BCG ANC Δ*pks1*-2. Both strains lack *pks1* as shown by Southern blot and do not produce PGL as tested by TLC (Fig. S6A–B). After infection of BMMs, both BCG ANC Δ*pks1*-1 and BCG ANC Δ*pks1*-2 survived significantly better compared with ANC after 3 and 6 days of infection (Fig. S6C). Thus, the genotypic and phenotypic changes of the SEL mycobacteria resulted in improved survival, not only in the macrophages where the selection was performed but also in primary macrophages. We then investigated the genes differentially expressed during re-infection that may contribute to improved survival. Using microarray analysis, we identified a distinct pattern of gene expression after re-infection with ANC versus SEL bacteria (Fig. S5). We confirmed by quantitative real-time PCR (qPCR) that the enzyme l-Lysine-epsilon aminotransferase (*lat*) and the resuscitation-promoter factor C (*rpfC)* were both significantly upregulated. On the other hand, the virulence-regulating protein *virS*, and the RNA polymerase sigma factor *sigI* were slightly or strongly downregulated respectively (Fig. 5D).

Finally, we tested SEL mycobacteria in a mouse model of infection. The ability of the SEL mycobacteria to survive improved significantly compared to the ancestral mycobacteria, since the numbers of selected bacteria in lungs of BALB/c mice were significantly higher after 35 days of infection (Fig. 5E), and the survival of Nude mice inoculated with the selected bacteria significantly decreased (*P* ≤ 0.0005, Fig. 5F). Additionally, we observed an increase in the number of bacteria when mice were infected with the BCG ANC Δ*pks1*-2 or SEL compared to ANC mycobacteria (Fig. S6D). These experiments strongly argue for an increased ability of the selected bacteria to remain in the mouse lungs and therefore validate our system using RAW 264.7 macrophages to select bacteria that are able to adapt to an *in vivo* system.

## Discussion

The aim of this study was to develop a system to understand the impact of a long-standing intracellular habitat for mycobacteria. Many of the *in vitro* studies of mycobacteria have been carried out in mouse primary macrophages. However, those cells can survive for a maximum of 2 weeks after isolation and are not suitable for long-term infections. In contrast, immortalized cell lines such as RAW 264.7 that have also been extensively used to analyse mycobacterial infections (Gutierrez *et al*., 2004), allow experiments to be carried out over relatively long periods of time. By monitoring the infection of RAW 264.7 macrophages with BCG we observed that mycobacteria were able to survive indefinitely in macrophage cultures. We found that one of the mechanisms of expansion of infected cells operating in the LTI culture was host cell death followed by efferocytosis (Martin *et al*., 2012). These observations suggest a role for macrophage efferocytosis in the maintenance of mycobacterial infection through a continuous population of recruited macrophages as previously described in other systems (Dannenberg, 2003; Cosma *et al*., 2004; Ramakrishnan, 2012). However, the process of infected cell expansion was rather stochastic, as some of the groups of infected cells expanded very well but others did not. Additionally, when cells were dividing, sometimes the phagosomes were also seen to divide, thus facilitating the transfer of bacteria to both daughter cells. Thus, SEL bacteria are also vertically transferred during the replication of host macrophages.

In LTI macrophages, mycobacteria reside in a hydrolytic compartment that has overcome the expected block in maturation. Since bacteria are viable, these data suggest that although SEL bacteria are exposed to a harsh environment, a sub-population seems to have adapted to it. It is known that mycobacteria have the capacity to resist acidic environments (Vandal *et al*., 2008; 2009[Bibr b43],[Bibr b44]). Our results are consistent with a scenario in which at the very early stages of infection, bacteria temporally block transport to lysosomes but later, after extended periods of time in macrophages, the blockage is released and bacteria adapt to a harsh environment, a feature that might be crucial for the establishment of a long-term infection. In agreement with this notion, it has been reported that mycobacteria internalized via efferocytosis are located in late endocytic compartments (Martin *et al*., 2012).

Our data showed that the SEL mycobacteria grow slower *in vitro* and have different metabolic features, including an increased capacity to use glucose as a carbon-source and an increased tendency for storage of neutral lipids in droplets, contributing to the survival of the bacteria intracellularly. Since the tendency of persistent mycobacteria to store fatty acids selectively in neutral lipids appears to be a consequence of an extended intracellular habitat, it is tempting to postulate that this change will result in an alteration of membrane fluidity in response to the intracellular stress as suggested for *Rhodococcus* (Alvarez and Steinbuchel, 2002). Although phenotypic variation has been traditionally linked to long-term bacterial adaptation to the host, genetic studies have also demonstrated a link between certain genes and long-term infections (Moyed and Bertrand, 1983; Moyed and Broderick, 1986; Chan *et al*., 2002; Lawley *et al*., 2006; Nguyen and Singh, 2006; Bianconi *et al*., 2011). Activation of different stress pathways might result not only in a change in phenotype but also in genetic adaptations (Beaumont *et al*., 2009; Ensminger *et al*., 2012).

*pks1* is part of the DIM+PGL locus, responsible for the synthesis of phenolic glycolipids (PGLs) and linked to the virulence of certain strains of *M. tuberculosis* (Reed *et al*., 2004; 2007[Bibr b35],[Bibr b36]; Sinsimer *et al*., 2008) and *M. leprae* (Tabouret *et al*., 2010). Based on our data, we concluded that the macrophage lifestyle has selected mycobacteria that have a non-functional *pks1*. It was unexpected to find a low level of change in the genetic composition after such a long period of time. However, *Mycobacterium* is a genus known for its low mutation rate in the host (Ford *et al*., 2011) and the high frequency of mutations in the *pks1/pks15* genetic region suggested that the trait(s) encoded by this region are under selective pressure (Pang *et al*., 2012). Moreover, single-nucleotide mutations can have highly pleiotropic effects on gene expression and phenotype that lead to adaptation (Brown *et al*., 2008; Beaumont *et al*., 2009). Although we cannot exclude undetected polymorphisms in specific promoter regions or transcription factors, our data suggest that variability in the *pks1/15* gene in the appropriate genomic and phenotypic context, contributes to the ability of mycobacteria to maintain a long-term intracellular infection. It is likely that additional mechanisms not investigated in this work, participate during this long-term selection in macrophages. These mechanisms include phenotypic changes that could have been lost during the re-isolation of bacteria *in vitro* and epigenetic changes via methylation (Shell *et al*., 2013). Therefore, further studies will be required to determine the contribution of those mechanisms to the long-term host cell adaptation.

Additionally, our approach has revealed a number of potential candidates regulated during host macrophage adaptation such as the enzyme l-Lysine-epsilon aminotransferase (lat) and the resuscitation-promoting factor C (rpfC). Interestingly, in many bacteria, *lat* has been associated with the ability to adapt to osmotic stress, starvation and the host cell environment (Betts *et al*., 2002; Gotz *et al*., 2010; Ensminger *et al*., 2012) and *rpfC* linked to the response to hypoxia (Gupta *et al*., 2010). The genes *virS* and *sigI* regulate multiple pathways, including resistance to stress (Estelmann *et al*., 2011; Homerova *et al*., 2012; Lee *et al*., 2012), and its differential expression may explain the ability of SEL to grow better in macrophages. Additional studies will clarify the precise function of these factors in the context of bacterial adaptation to the host cells. An important fact to consider is that we used BCG as a model organism. However, this bacterium has lost several genetic regions important for the virulence of *M. tuberculosis* such as the RD1 region (Hsu *et al*., 2003). Another crucial question that remains to be addressed is how closely our model correlates to the intracellular state of *M. tuberculosis* in an *in vivo* long-term infection in humans.

In summary, we describe in this report a cell-based approach that allows selection of long-term intracellular bacteria. Bacteria selected using this approach, revealed some striking metabolic changes, including lack of PGL production, higher accumulation of neutral lipids, improved ability to metabolize glucose and differential expression of specific genes in response to intracellular environment. Understanding the impact of evolutionary forces imposed by the host on their bacterial guests during long-term infections is critical in our understanding of recalcitrant bacterial infections. However, the basis of the adaptations to cellular hosts is ill defined. In the last few years, different studies have addressed the experimental evolution of bacteria *in vitro* for long periods of time (Elena and Lenski, 2003) and more recently, also in intracellular systems, albeit for relatively short times (Ensminger *et al*., 2012; Rohde *et al*., 2012). Our described model introduces a system that makes it possible to follow the process of bacterial adaptation to the cell host for many years. We foresee that this strategy of long-term bacterial selection through cell culture, is not only applicable to mycobacteria, but may also be applicable to other intracellular pathogens, such as *Chlamydia*, *Brucella* and *Salmonella*. Systems that reduce complexity in defined environments like such as the one described here represent an invaluable tool to study fundamental processes of bacterial adaptations to a host cell.

## Experimental procedures

### Cells

RAW 264.7 macrophages were obtained from the American Type Culture Collection (ATCC, Cat# TIB-71) and maintained in complete Dulbecco's Modified Eagles Medium (D-MEM) with 4.5 g l^−1^ glucose, 10% (v/v) heat-inactivated fetal calf serum (FCS, PAA, Austria) and 2 mM l-glutamine (PAA, Austria) (complete D-MEM medium). Bone marrow macrophages (BMMs) were isolated and maintained as described previously (Kasmapour *et al*., 2012). Cells were incubated at 37°C and 5% CO_2_ in a humidified incubator. *M. bovis* BCG str. Pasteur 1173P2 expressing GFP (BCG-GFP, ancestral strain (ANC) kindly provided by Dr Brigitte Gicquel, Pasteur Institute, France) were grown in roller flasks at 37°C in Middlebrook 7H9 liquid medium (Difco Laboratories, USA) containing 0.2% glycerol, 0.05% Tween 80 and supplemented with 10% OADC (oleic acid-albumin-dextrose-catalase supplement) (BD Biosciences, USA) or in Middlebrook 7H10 plates (Difco Laboratories, USA) supplemented with 10% OADC.

### Generation of long-term infected macrophages (LTI)

RAW 264.7 macrophages were grown in T-75 flasks (Marienfeld GmbH & Co, Germany) at approximately 80% confluence and were infected with ANC at MOI of 10. After 24 h, cells were washed with phosphate-buffered saline (PBS) three times and incubated at 37°C in 5% CO_2_ atmosphere. At this point, several aliquots of the ancestral strain were stored at −80°C. For the subsequent passages: after 2–3 days, cells reached confluence and needed splitting. For that, cells were scraped and were carefully resuspended in 15 ml of complete D-MEM and 4 ml of the suspension was transferred to a new T-75 flask for incubation. After several weeks of infection, cells grew slower and needed splitting after 5 days. Then, every 5 days, the infected cell cultures were split; every 10 days (two passages), samples were lysed and frozen for colony-forming unit (cfu) analysis. Unless otherwise stated, the following standards were maintained: (i) the liquid culture medium was D-MEM 4.5 g l^−1^ glucose supplemented with 10% heat-inactivated FCS and 2.5 mM of l-glutamine (complete medium), (ii) cells were seeded on T-75 flasks, and (iii) infected cultures were incubated at 37°C, 5% CO_2_ in a humidified incubator.

### RAW 264.7 macrophages recently infected (RI)

RAW 264.7 macrophages seeded on T-75 flasks or on 24-well plates (Marienfeld GmbH & Co, Germany) were infected with ANC (MOI: 2.5) and resuspended in complete D-MEM medium. After 2 h of uptake, cells were washed three times with PBS and fresh medium was added. Cells were incubated during 24 h at 37°C in 5% CO_2_ atmosphere. Macrophages infected with ANC for 24 h were defined as ‘recently infected’ (RI).

### cfu determination

Cells were lysed in 1 ml of sterile water and frozen for cfu analysis. Lysates were serially diluted in PBS-Tween 80 0.05% (Sigma, Germany) and plated onto complete 7H10 agar medium. Colony-forming units were determined as the mean of three plates at each time point after 3 or 4 weeks.

### Live cell imaging and image analysis

For live cell imaging, 1 × 10^5^ long-term infected macrophages were seeded on 35 mm glass bottom dishes (MatTek, USA). Cells were washed with PBS and replaced with imaging medium: complete D-MEM medium without phenol Red, shortly before imaging with a Leica SP5 AOBS Laser Scanning Confocal Microscope (Leica Microsystems, Germany) equipped with an environment control chamber (EMBLEM, Germany). During imaging, a single focal plane was monitored in time (xyt scanning mode) using a 63×/1.4 HCX-PLAPO oil objective, an Argon Laser (488 nm) and DPSS Laser (561 nm) when applicable, scanner frequency 200–400 Hz; line averaging 2–3, at a rate of 7–15 s per frame using PMT and/or HyD detectors at a scanning resolution of 1024 × 1024 pixels. Analysis was performed using ImageJ (US National Institute of Health, Bethesda, Maryland, USA) and Fiji. Fiji is a distribution of ImageJ available at http://fiji.sc. Iterative versions of ImageJ used for this work are 1.41m through 1.46a. For analysis, RGB images or frames were split into separate colour channels. Bacteria (green channel) were thresholded per single cell and the fluorescence intensity of the marker of interest associated to the phagosome was measured for each frame by re-directing measurements to the channel of interest. Fluorescence intensity values were plotted using GraphPad Prism software (GraphPad Software, USA).

### Flow cytometry

In order to analyse the total amount of infected cells in the LTI macrophages, LTI cells were scraped and resuspended in PBS with 1% BSA (Albumin bovine serum, Sigma, Germany). Cells were counted and 6 × 10^6^ cells ml^−1^ were analysed using a LSRII (BD Bioscience, CA, USA). Data obtained were evaluated using FACS DiVA (BD Bioscience, CA, USA) or FlowJo Mac v8.84 (Tree Star, Ashland, OR, USA). To analyse cell death, LTI or RI macrophages were scraped and resuspended in PBS with 1% BSA (Albumin bovine serum, Sigma, Germany). Cells were counted and 5 × 10^6^ cells ml^−1^ were resuspended in binding buffer (0.01 M Hepes pH 7.4, 0.14 M NaCl, 0.25 mM CaCl_2_). Cells were stained with APC Annexin V (BD Biosciences, CA, USA) and 7-AAD (7-amino-actinomycin D, BD Biosciences, CA, USA) following the manufacturer's instructions, to distinguish apoptotic and dead/necrotic cells. After staining, cells were stored on ice until analysis. As a control for apoptotic cell death, uninfected RAW 264.7 cells were exposed to UV light for 15 min and incubated for 6 h before analysis. Samples were analysed using a LSRII (BD Bioscience, CA, USA) and data obtained were evaluated using FACS DiVA (BD Bioscience, CA, USA) or FlowJo Mac v8.84 (Tree Star, Ashland, OR, USA).

### Efferocytosis by live cell imaging

To visualize efferocytosis, 3 × 10^4^ LTI macrophages were seeded on a 35 mm glass bottom dish (MatTek, USA) and incubated for 8 h. Then cells were washed with PBS and replaced with imaging medium. Afterwards, 1 × 10^4^ uninfected RAW 264.7 macrophages stably overexpressing ER-mCherry were added to the dish and after 4 h (to allow the uninfected cells to attach) the imaging was started. One of the groups of infected cells was localized and a single focal plane and monitored in time (xyt scanning mode), every 5 min for approximately 4 days. The efferocytosis process was observed in infected cells that die by apoptosis and then are internalized by another cell.

### Indirect immunofluorescence

A total of 1 × 10^5^ RI and LTI cells seeded on 24-well plates with coverslips (1 × 10^5^ cells per well) (Marienfeld GmbH & Co, Germany) were fixed at room temperature with 3% PFA/PBS pH 7.4 (Electron Microscopy Science, USA) for 10 min followed by 50 mM glycin/PBS pH 7.4 (Sigma, Germany) for 10 min. Then cells were permeabilized with 0.05% saponin (Fluka, Germany), 1% BSA (Sigma, Germany) in PBS for 10 min. Following incubation with the primary and secondary antibodies diluted in PBS, DNA staining (10 min in 1 μg ml^−1^ Hoechst 33258 in PBS, Sigma, Germany) was performed. Coverslips were mounted on slides with DAKO mounting medium (Dako Cytomation, Denmark). A polyclonal rabbit anti-LC3 antibody (MBL, Woburn, MA, USA) was used 1:300 overnight in a humidified chamber. Rat anti-LAMP-2 (DSHB, Iowa, USA) was used 1:50 for 1 h at room temperature. Secondary antibodies were used according to manufacturer's instructions (Jackson ImmunoResearch, PA, USA). Actin was detected incubating the cells with 0.22 nM Rhodamine-phalloidin (Invitrogen, USA) in PBS for 10 min. Samples were analysed using a Leica SP5 AOBS Laser Scanning Confocal Microscope (Leica Microsystems, Germany).

### Lysotracker and DQ-BSA staining

RI and LTI cells seeded on coverslips (Marienfeld GmbH & Co, Germany) were labelled for 30 min at 37°C with a 50 nM Lysotracker Red solution. Self-quenched BODIPY dye conjugated to bovine serum albumin labelling was performed (DQ-BSA; Molecular Probes, Invitrogen, USA), in order to measure lysosomal function. Cells were pre-incubated for 1 h at 37°C with 10 μg ml^−1^ DQ-BSA in PBS. Then, cells were fixed at room temperature with 3% PFA in PBS pH 7.4 (Electron Microscopy Science, USA) for 10 min followed by 50 mM glycine in PBS pH 7.4 (Sigma, Germany) for 10 min.

### Isolation of selected bacteria *in vitro*

In order to re-isolate the selected BCG-GFP (SEL) *in vitro*, LTI macrophages were lysed in sterile water and plated onto Middlebrook 7H10 agar medium supplemented with 10% OADC. The plates were incubated at 37°C in 5% CO_2_ atmosphere. After 3 or 4 weeks, some of the grown colonies were transferred to Middlebrook 7H9 medium supplemented with 10% OADC, 0.2% glycerol, 0.05% Tween 80 (complete medium) and 50 μg ml^−1^ hygromycin (Roth, Germany). SEL was incubated for about 2 weeks at 37°C in a 5% CO_2_ atmosphere. At this point, several aliquots of the SEL were stored at −80°C. In order to maintain the features of the SEL bacteria, new SEL bacteria were thawed and used for all the *in vitro* experiments (passage 1, 2 or 3).

### Bacterial growth *in vitro*

In complete medium: samples of SEL and ANC bacteria with OD_600_: 0.1 were grown *in vitro* in Middlebrook 7H9 complete medium plus hygromycin 50 μg ml^−1^. Bacteria were incubated for 10 days and a small aliquot of SEL or ANC culture was taken every day in order to measure the OD_600_ to calculate the growth rate of the different bacteria. An Eppendorf Biophotometer Spectrophotometer (HA, Germany) was used for the measurements.

In Sauton medium: samples of SEL and ANC bacteria from an *in vitro* culture with OD_600_: 0.1 were grown in Sauton medium (KH_2_PO_4_ 0.5 g, MgSO_4_×7H_2_O 0.5 g, citric acid 2.0 g, ferric ammonium citrate 0.05 g, glycerol 60 ml, asparagine 4.0 g, pH 7.4) supplemented with 0.03% Tween 80, 6% glycerol or 1% glucose. To calculate the percentage of bacterial growth, the OD of the culture was measured at 570 nm after 7 and 14 days of incubation at 37°C using a Synergy 2 Multi-Detections-Reader (Biotek, USA).

### Mouse infections

Animal experiments were performed inside the biosafety facilities of the National Institute of Agricultural Technology (INTA), Argentina, in compliance with the regulations of Institutional Animal Care and Use Committee (CICUAE) of INTA. Ethical approval for the study was obtained from CICUAE (No. 34/2012). Groups of five female BALB/c mice (6–8 weeks old) were intratracheally inoculated with 1.25 × 10^5^ cfu of ANC, SEL mycobacteria or BCG Δ*pks1*-2. At 1 and 35 days post infection, lungs were removed rapidly after sacrifice and cfu counts were performed using the diluted organ homogenates. This experiment was repeated twice with similar results. Student's *t*-test was used to determine the statistical significance of cfu, a *P* ≤ 0.01 was considered significant. Groups of 10 immunodeficient Nude (*Foxn1^nu^*) female mice (6–8 weeks old) were infected with either ANC or SEL mycobacteria by intratracheal instillation of 1.25 × 10^4^ cfu. Statistical analysis for survival curves was performed using Mantel-Cox tests, *P* ≤ 0.0005.

### Statistical analysis

Statistical calculations and normalizations were performed using GraphPad Prism software version 5.0a (GraphPad Software, USA). *P*-values were calculated using Student's two tailed *t*-test as indicated. For the survival experiment in mice Mantel-Cox tests was used.

## Supporting Information

Supp. material and methods.

**Fig. S1.** Mycobacteria in LTI macrophages are mostly intracellular. A and B. LTI cultures of macrophages were plated in presence or absence of 10 μg ml^−1^ gentamicin as indicated in the panel (A). Macrophages were lysed and plated at the indicated time points and cfu were calculated (B). No differences were observed when gentamicin was present to kill extracellular bacteria indicating that during this period of time, bacteria were within macrophages and protected from the antibiotic. Data represent the mean ± S.E.M. of at least three independent experiments. C. Images showing Middlebrook 7H10 complete agar medium plates inoculated with the supernatant or lysed of LTI cells after centrifugation at 150 *g*, in order to check the presence of extracellular bacteria in the LTI culture untreated with gentamicin. D. Quantification of 15 replicates of the supernatant and lysed samples of LTI macrophages in the plates shown in (C) after 4 weeks of incubation. Data represent the mean ± S.E.M. of at least three independent experiments. E. Quantitative analysis of the green fluorescence intensity associated to a single macrophage in the long-term infected culture during 48 h. LTI macrophages were plated and analysed by live cell imaging for 2 days after plating. The plot is representative of one macrophage from 25 analysed.

**Fig. S2.** Mycobacteria in the LTI macrophages are localized in autophagic compartments and accumulate neutral lipids in droplets. A and B. Electron microscopy of LTI RAW 264.7 cells. Micrographs show the intracellular location of mycobacteria in late endocytic/autophagic compartments. Asterisks: selected mycobacteria. Scale bar: 1μm. C and D. Mycobacterial lipid droplets in LTI macrophages. Micrographs show the dramatic accumulation of intracellular lipid droplets in mycobacteria from LTI cultures. Scale bar: 1 μm (C), 200 nm (D, inset; asterisks: lipid droplets). E. LTI macrophages were stained with Nile red and observed by confocal microscopy. Both, macrophages (main panel) and bacteria (E′, inset) showed positive staining for Nile red in droplets. Nu: nucleus. Scale bar: 10 μm. F. Quantitative analysis of Nile red fluorescence associated to mycobacteria in RI and LTI macrophages. Data represent the mean ± S.E.M. of three independent experiments with at least 20 bacteria counted per condition, (**) *P* ≤ 0.01 from two-tailed Student's *t*-test.

**Fig. S3.** Lipid dynamics of ANC versus SEL mycobacteria *in vitro*. Isotopic labelling and incorporation of fatty acids associated to phospholipids (PL), glycolipids (GL) and neutral lipids (NL) in selected mycobacteria *in vitro* during 13 days of incorporation of ^13^C-acetate in 7H9 complete medium.

**Fig. S4.** Genotypic changes of SEL. A. Diagram showing the strategy followed to map and compare the genomes of ANC and SEL mycobacteria. B. PGL production in ancestral and selected mycobacteria. Extraction of glycolipids and thin-layer chromatography (TLC) were performed in supernatants and pellets of ANC versus SEL mycobacteria. Samples were loaded by duplicates. Image is representative of more than three independent experiments.

**Fig. S5.** Gene expression analysis during re-infection with ANC and SEL by microarray. Heat map of the genes analysed by microarray. Three independent samples were analysed per duplicate in two different microarrays. The *P*-value cut-off was 0.05 and the fold-change cut-off ≥ 2.0. After analysis, 38 genes were differentially regulated (either up- or downregulated), of them 14 were analysed in duplicates or triplicates. Blue and red squares indicate genes down- or upregulated in SEL respectively that were analysed by qPCR.

**Fig. S6.** A. Southern blot analysis of DNA samples digested with PstI and probed with nucleotide 3002 to nucleotide 3248 of *pks1*. Lanes 1 and 3: ANC Δ*pks1*-1 and ANC BCG Δ*pks1*-2 clones. Lane 2: ANC strain. Arrow indicates the band corresponding to *pks1* and other bands correspond to homologous to *pks1* on BCG genome. B. PGL production in ANC, SEL, ANC Δ*pks1*-1 and ANC Δ*pks1*-2 clones. Extraction of glycolipids and thin-layer chromatography (TLC) were performed in pellets of the different strains of mycobacteria. Image is representative of more than three independent experiments. C. ANC, ANC Δ*pks1*-1 and ANC Δ*pks1*-2 clones were used to re-infect primary bone marrow macrophages (BMMs) as indicated above. After 1, 3 and 6 days of infection, the number of colonies was counted and cfu were calculated. Data represent the mean ± S.E.M. of three independent experiments, (*) *P* ≤ 0.05, (**) *P* ≤ 0.01 and (***) *P* ≤ 0.001 from two-tailed Student's *t*-test. D. Lung bacillary loads in BALB/c mice after intratracheal inoculation (1.25 × 10^5^ cfu) of ancestral (ANC, green bars), selected (SEL, red bars) or Δ*pks1* BCG (ANC Δpks1-2, blue bars) bacteria The data represent the mean number of cfu ± SD in five mice of one representative experiment out of two independent experiments, (*) *P* ≤ 0.05 and (**) *P* ≤ 0.01 from two-tailed Student's *t*-test.

**Table S1.** Single-nucleotide polymorphisms (SNP) pair-wise comparison of ANC versus SEL mycobacteria. For the SNP analysis 18 single-nucleotide differences (coverage > 20) could be found between the two strains and the reference, but no significant differences between them were found.

**Table S2.** Deletion insertion polymorphisms (DIP) comparison of ANC versus SEL mycobacteria.

**Movie S1.** Expansion of the infection in LTI macrophages. LTI macrophages images were acquired throughout the indicated period (38.5 h). Each image was captured every 3.45 min. This video corresponds to Fig. 2A.

**Movie S2.** Propagation of the infection in LTI macrophages showing cell death, bacterial growth and efferocytosis. Images were acquired during 62 h and each image was acquired every 5 min. This video corresponds to Fig. 2D.

**Movie S3.** Cell division of LTI macrophages. Images from long-term infected macrophages were captured throughout the indicated period of time (37.5 h). The acquisition of each image was every 40 s. This video corresponds to Fig. 2E.

## References

[b1] Alvarez HM, Steinbuchel A (2002). Triacylglycerols in prokaryotic microorganisms. Appl Microbiol Biotechnol.

[b2] An FQ, Folarin HM, Compitello N, Roth J, Gerson SL, McCrae KR (2006). Long-term-infected telomerase-immortalized endothelial cells: a model for Kaposi's sarcoma-associated herpesvirus latency *in vitro* and *in vivo*. J Virol.

[b3] Beaumont HJ, Gallie J, Kost C, Ferguson GC, Rainey PB (2009). Experimental evolution of bet hedging. Nature.

[b4] Betts JC, Lukey PT, Robb LC, McAdam RA, Duncan K (2002). Evaluation of a nutrient starvation model of *Mycobacterium tuberculosis* persistence by gene and protein expression profiling. Mol Microbiol.

[b5] Bianconi I, Milani A, Cigana C, Paroni M, Levesque RC, Bertoni G, Bragonzi A (2011). Positive signature-tagged mutagenesis in *Pseudomonas aeruginosa*: tracking patho-adaptive mutations promoting airways chronic infection. PLoS Pathog.

[b6] Boshoff HI, Barry CE (2005). Tuberculosis – metabolism and respiration in the absence of growth. Nat Rev Microbiol.

[b7] Brown KM, Landry CR, Hartl DL, Cavalieri D (2008). Cascading transcriptional effects of a naturally occurring frameshift mutation in *Saccharomyces cerevisiae*. Mol Ecol.

[b8] Casadevall A (2008). Evolution of intracellular pathogens. Annu Rev Microbiol.

[b9] Chan K, Knaak T, Satkamp L, Humbert O, Falkow S, Ramakrishnan L (2002). Complex pattern of *Mycobacterium marinum* gene expression during long-term granulomatous infection. Proc Natl Acad Sci USA.

[b10] Cosma CL, Humbert O, Ramakrishnan L (2004). Superinfecting mycobacteria home to established tuberculous granulomas. Nat Immunol.

[b11] Dannenberg AM (2003). Macrophage turnover, division and activation within developing, peak and ‘healed’ tuberculous lesions produced in rabbits by BCG. Tuberculosis (Edinb).

[b12] Eisenreich W, Dandekar T, Heesemann J, Goebel W (2010). Carbon metabolism of intracellular bacterial pathogens and possible links to virulence. Nat Rev Microbiol.

[b13] Elena SF, Lenski RE (2003). Evolution experiments with microorganisms: the dynamics and genetic bases of adaptation. Nat Rev Genet.

[b14] Ensminger AW, Yassin Y, Miron A, Isberg RR (2012). Experimental evolution of *Legionella pneumophila* in mouse macrophages leads to strains with altered determinants of environmental survival. PLoS Pathog.

[b15] Estelmann S, Hugler M, Eisenreich W, Werner K, Berg IA, Ramos-Vera WH (2011). Labeling and enzyme studies of the central carbon metabolism in *Metallosphaera sedula*. J Bacteriol.

[b16] Flannagan RS, Cosio G, Grinstein S (2009). Antimicrobial mechanisms of phagocytes and bacterial evasion strategies. Nat Rev Microbiol.

[b17] Ford CB, Lin PL, Chase MR, Shah RR, Iartchouk O, Galagan J (2011). Use of whole genome sequencing to estimate the mutation rate of *Mycobacterium tuberculosis* during latent infection. Nat Genet.

[b18] Fritz C, Maass S, Kreft A, Bange FC (2002). Dependence of *Mycobacterium bovis* BCG on anaerobic nitrate reductase for persistence is tissue specific. Infect Immun.

[b19] Gotz A, Eylert E, Eisenreich W, Goebel W (2010). Carbon metabolism of enterobacterial human pathogens growing in epithelial colorectal adenocarcinoma (Caco-2) cells. PLoS ONE.

[b20] Gupta RK, Srivastava BS, Srivastava R (2010). Comparative expression analysis of rpf-like genes of *Mycobacterium tuberculosis* H37Rv under different physiological stress and growth conditions. Microbiology.

[b21] Gutierrez MG, Master SS, Singh SB, Taylor GA, Colombo MI, Deretic V (2004). Autophagy is a defense mechanism inhibiting BCG and *Mycobacterium tuberculosis* survival in infected macrophages. Cell.

[b22] Homerova D, Sevcikova B, Rezuchova B, Kormanec J (2012). Regulation of an alternative sigma factor sigmaI by a partner switching mechanism with an anti-sigma factor PrsI and an anti-anti-sigma factor ArsI in *Streptomyces coelicolor* A3(2). Gene.

[b23] Honer zu Bentrup K, Russell DG (2001). Mycobacterial persistence: adaptation to a changing environment. Trends Microbiol.

[b9001] Hsu T, Hingley-Wilson SM, Chen B, Chen M, Dai AZ, Morin PM (2003). The primary mechanism of attenuation of bacillus Calmette-Guerin is a loss of secreted lytic function required for invasion of lung interstitial tissue. Proc Natl Acad Sci.

[b24] Kasmapour B, Gronow A, Bleck CK, Hong W, Gutierrez MG (2012). Size-dependent mechanism of cargo sorting during lysosome–phagosome fusion is controlled by Rab34. Proc Natl Acad Sci USA.

[b25] Lawley TD, Chan K, Thompson LJ, Kim CC, Govoni GR, Monack DM (2006). Genome-wide screen for *Salmonella* genes required for long-term systemic infection of the mouse. PLoS Pathog.

[b26] Lee JH, Ammerman NC, Nolan S, Geiman DE, Lun S, Guo H, Bishai WR (2012). Isoniazid resistance without a loss of fitness in *Mycobacterium tuberculosis*. Nat Commun.

[b27] Leversen NA, Sviland L, Wiker HG, Mustafa T (2012). Long-term persistence of BCG Pasteur in lungs of C57BL/6 mice following intranasal infection. Scand J Immunol.

[b28] Martin CJ, Booty MG, Rosebrock TR, Nunes-Alves C, Desjardins DM, Keren I (2012). Efferocytosis is an innate antibacterial mechanism. Cell Host Microbe.

[b29] Monack DM, Mueller A, Falkow S (2004). Persistent bacterial infections: the interface of the pathogen and the host immune system. Nat Rev Microbiol.

[b30] Moyed HS, Bertrand KP (1983). *hipA*, a newly recognized gene of *Escherichia coli* K-12 that affects frequency of persistence after inhibition of murein synthesis. J Bacteriol.

[b31] Moyed HS, Broderick SH (1986). Molecular cloning and expression of *hipA*, a gene of *Escherichia coli* K-12 that affects frequency of persistence after inhibition of murein synthesis. J Bacteriol.

[b32] Nguyen D, Singh PK (2006). Evolving stealth: genetic adaptation of *Pseudomonas aeruginosa* during cystic fibrosis infections. Proc Natl Acad Sci USA.

[b33] Pang JM, Layre E, Sweet L, Sherrid A, Moody DB, Ojha A, Sherman DR (2012). The polyketide Pks1 contributes to biofilm formation in *Mycobacterium tuberculosis*. J Bacteriol.

[b34] Ramakrishnan L (2012). Revisiting the role of the granuloma in tuberculosis. Nat Rev Immunol.

[b35] Reed MB, Domenech P, Manca C, Su H, Barczak AK, Kreiswirth BN (2004). A glycolipid of hypervirulent tuberculosis strains that inhibits the innate immune response. Nature.

[b36] Reed MB, Gagneux S, Deriemer K, Small PM, Barry CE (2007). The W-Beijing lineage of *Mycobacterium tuberculosis* overproduces triglycerides and has the DosR dormancy regulon constitutively upregulated. J Bacteriol.

[b37] Rohde KH, Veiga DF, Caldwell S, Balazsi G, Russell DG (2012). Linking the transcriptional profiles and the physiological states of *Mycobacterium tuberculosis* during an extended intracellular infection. PLoS Pathog.

[b38] Shell SS, Prestwich EG, Baek SH, Shah RR, Sassetti CM, Dedon PC, Fortune SM (2013). DNA methylation impacts gene expression and ensures hypoxic survival of *Mycobacterium tuberculosis*. PLoS Pathog.

[b39] Sinsimer D, Huet G, Manca C, Tsenova L, Koo MS, Kurepina N (2008). The phenolic glycolipid of *Mycobacterium tuberculosis* differentially modulates the early host cytokine response but does not in itself confer hypervirulence. Infect Immun.

[b40] Stewart GR, Robertson BD, Young DB (2003). Tuberculosis: a problem with persistence. Nat Rev Microbiol.

[b41] Sturgill-Koszycki S, Schlesinger PH, Chakraborty P, Haddix PL, Collins HL, Fok AK (1994). Lack of acidification in *Mycobacterium* phagosomes produced by exclusion of the vesicular proton-ATPase. Science.

[b42] Tabouret G, Astarie-Dequeker C, Demangel C, Malaga W, Constant P, Ray A (2010). *Mycobacterium leprae* phenolglycolipid-1 expressed by engineered *M. bovis* BCG modulates early interaction with human phagocytes. PLoS Pathog.

[b43] Vandal OH, Pierini LM, Schnappinger D, Nathan CF, Ehrt S (2008). A membrane protein preserves intrabacterial pH in intraphagosomal *Mycobacterium tuberculosis*. Nat Med.

[b44] Vandal OH, Roberts JA, Odaira T, Schnappinger D, Nathan CF, Ehrt S (2009). Acid-susceptible mutants of *Mycobacterium tuberculosis* share hypersusceptibility to cell wall and oxidative stress and to the host environment. J Bacteriol.

[b45] Via LE, Fratti RA, McFalone M, Pagan-Ramos E, Deretic D, Deretic V (1998). Effects of cytokines on mycobacterial phagosome maturation. J Cell Sci.

[b46] Wang L, Damania B (2008). Kaposi's sarcoma-associated herpesvirus confers a survival advantage to endothelial cells. Cancer Res.

