# Supplementary material for: Experimental selection of long-term intracellular mycobacteria
Source: Cell Microbiol. 2014 Jun 2;16(9):1425–40. doi: 10.1111/cmi.12303 (PMC4283733; doi:10.1111/cmi.12303)
Supplement: Supplementary file 1 — Supp. material and methods. [file cmi0016-1425-sd1.docx]

**Supplementary Material for:**

**Experimental selection of long-term intracellular mycobacteria**

**Vazquez et al.**

**Supp. material and methods**

**Thin-layer chromatography (TLC)**

Thin layer chromatography was performed as described in Constant et al. 2002 (1). ANC and SEL mycobacteria were separated from the culture media and both were kept. The media were sterilized by filtration through 0.2 µm pore size membrane and concentrated to one-tenth of the initial volume. Cells were left in CHCl_3_/CH_3_OH, 2:1, v/v for 2 days at room temperature to kill bacteria. Lipids were extracted twice with CHCl_3_/CH_3_OH, 1:1, v/v, washed twice with water and dried. Lipids from culture media were obtained by adding 2 volumes of CH_3_OH and 1 volume of CHCl_3_ to 0.8 volume of concentrated culture medium to yield a homogenous one-phase mixture. After a 2 h period, the mixture was partitioned into two phases by the addition of 1 volume of H_2_O/CHCl_3_, 1:1, v/v. The organic phases were recovered, washed twice with water, and dried. The production of PGL by the ancestral and selected strains was analyzed by thin-layer chromatography (TLC). Briefly, the extracts were dissolved in CHCl_3_ to give a final lipid concentration of 20 mg/mL. Equivalent volumes of each extract were deposited on silica gel G 60 plates (20x20 cm, Merck), which were run in CHCl_3_/CH_3_OH (95:5, v/v) for the detection of PGLs. Glycolipids were visualized by spraying the plates with 0.2 % anthrone (w/v) in concentrated H_2_SO_4_, followed by heating.

**Stable isotope probing**

ANC or SEL mycobacteria were grown in Middlebrook 7H9 media and 1 mg of one of two different ^13^C-labelled carbon sources were additionally added at day 0. ANC and SEL bacteria were grown at 37 °C and 100 r.p.m. in 2 L flasks filled with 200 mL medium. The biomass was harvested after 0, 1, 3, 6, 9, and 13 days. The starting OD_600_ of all experiments was 0.1. All experiments were performed and analyzed twice in all media.

**Polar lipid fatty acid analysis**

Lipids were extracted from the biomass using a modified Bligh-Dyer procedure (2) as described in Vancanneyt et al. 1996 (3). The total lipid fraction was fractionated into polarity classes neutral lipids (NL), glycolipids (GL) and phospholipids (PL) and the fatty acid methyl esters (FAMEs) prepared as described previously (4). Individual fatty acid methyl esters were identified by mass spectra and their retention time using standards. For comparison the wet biomass was directly saponified to get the cellular fatty acids using the MIDI protocol (5).

**GC/C/IRMS**

Measurements were performed on a Finnigan MAT 253 isotope ratio mass spectrometer in triplicate. It was coupled via a combustion interface with a HP 5890 gas chromatograph. The fatty acid methyl esters were separated with a Restek Rtx-2 column (60 m, 0.32 mm inner diameter, 0.25 μm film thickness). The injector temperature was set to 250 °C. The column effluent was combusted on-line in an oxidation oven (copper, nickel, platinum-catalyst, 980 °C), passed through a reactor with elemental copper (600 °C) for reducing NO. Combustion gas was dried by a water-permeable membrane (Nafion).

**Notation**

The standard notation for expression of high-precision gas isotope ratio mass spectrometry results in the δ notation defined as:

δ (‰) = ((R_FAME_/R_PDB_)-1)x10^3^ (2)

where R_FAME_ and R_PDB_ are the ^13^C/^12^C isotope ratios corresponding respectively to the sample and to the international internal standard PeeDee Belemnite, a South Carolinian carbonate rich in ^13^C (R_PDB_ = 0.0112372 ± 0.0000090) (6).

**Calculation of the isotope ratios of the fatty acids**

The esterification of the fatty acids with methanol introduced one additional carbon which was not present in the parent compound altering the original isotope ratio of the fatty acids. However, the derivatization process introduced a distinct reproducible fractionation that was constant for each fatty acid allowing the calculation of the isotope ratio of the free fatty acids (7, 8). This was done by using the formula:

δ^13^C_FA_ = ((C_n_+1)xδ^13^C_FAME_‑δ^13^C_MeOH_)/C_n_

δ^13^C_FA_ = δ^13^C of the fatty acid

C_n_ = number of carbons in the fatty acid

δ^13^C_FAME_ = δ^13^C of the fatty acid methyl ester (FAME)

δ^13^C_MeOH_ = δ^13^C of methanol used for the methylating reaction = -37.6 %

to calculate the isotope ratios of the fatty acids (9, 10).

**Library construction and re-sequencing**

ANC and SEL mycobacteria were grown in complete Middlebrook 7H9 medium for 24 h and the DNA was isolated using DNA isolation kit (FastDNA™ SPIN Kit, MP Biomedicals, CA, USA). Library and sequencing using Illumina GAII sequencer were performed by BaseClear BV (Leiden, The Netherlands). High quality DNA was provided and its quality analyzed on the Bioanalyser. The DNA was fragmented; size selected and the ends of the sheared molecules blunted. Multiplex DNA adapters were added to both ends of the DNA fragments and PCR amplification was performed on the molecules using the added adapter regions as priming sites. The resultant product was loaded on the Genome analyzer.

**Bioinformatics analysis**

SNP (single nucleotide polymorphism) analysis of both ANC and SEL against the reference sequence and between each other was performed. Briefly, the length of the reference BCG Pasteur 1173P2 (NC_008769) is 4,374,522 nucleotides. For ANC bacteria the Illumina reads could cover 4,203,858 of these positions and for SEL mycobacteria 4,204,085 nucleotides, assemblies in FASTA format, with N’s at positions without coverage (gaps). SNP analysis was performed for positions with a coverage >4 to be as complete as possible, however some significance could only be given for SNPs in the table that have a coverage of at least >20. DIP (small nucleotide deletion-insertion) analysis (coverage >20) yielded three DIPs between ANC and the reference BCG NC_008769, and four DIPs between SEL and BCG NC_008769. In order to validate the insertion of a guanine nucleotide that generated a frameshift in the *pks1* gene of the SEL bacteria, 10 individual colonies of SEL bacteria were isolated and growth in complete Middlebrook 7H9 medium and the DNA of each one was isolated using DNA isolation kit (FastDNA™ SPIN Kit, MP Biomedicals, CA, USA). PCR reaction was done to amplify the *pks1* gene using the following primers: -F: 5'-CGCGTCATTTGCAGACCCTTGGG-3’, -R: 5'-GTGCTTTCGCTCGCCTCAACC-3' (5 PRIME HotMaster *Taq* DNA Polymerase kit, Hilden, Germany). The presence of the insertion was confirmed by sequencing (Eurofins MWG Operon, Germany).

**Re-infection of RAW 264.7 and BMM with ANC and SEL bacteria**

ANC and SEL mycobacteria were used to re-infect RAW 264.7 or Bone marrow macrophages (BMM) (MOI: 2.5) in complete D-MEM medium for RAW 264.7 cells, and D-MEM supplemented with 10 % FCS, L-glutamine 2.5 mM, 5 % Horse serum (PAA, Austria) and 20 % L929 cells supernatant for BMM cells. After 1 h of uptake, cells were washed 3 times with PBS and fresh medium plus gentamicin 10 µg/mL (Sigma, Germany) was added. Cells were incubated for 1, 2, 3 or 7 days at 37 °C in 5 % CO_2_ atmosphere. Subsequently, cells were lysed in sterile water and serially diluted in PBS-Tween 80 0.05 %. Dilutions were plated in Middlebrook 7H10 complete agar medium. Colonies were counted after 3 to 4 weeks to determine CFU.

**RNA isolation from intracellular bacteria**

We used the guanidinium thiocyanate (GTC)-based lysis solution method to extract total RNA from intracellular mycobacteria (11). Briefly, macrophages seeded at 60 % of confluence on 20 T-150 flasks (Marienfeld GmbH & Co, Germany) were infected with ANC or SEL bacteria (MOI: 2.5) in complete D-MEM medium for 24 h. The infected monolayers were resuspended in GCT solution (4 M GTC, 0.5 % sodium N-lauryl sarcosine, 25 mM tri-sodium citrate, 0.1 M 2-mercaptoethanol, 0.5 % Tween 80, pH 7.0 (All the reagents were from Sigma, Germany). The bacteria in the GCT solution were centrifuged and the bacterial pellet was disrupted using FastRNA® Pro Blue kit from Q-BIOgene (MP Biomedicals, CA, USA) following the manufacturer´s instructions. Mycobacterial RNA was extracted with the RNeasy mini kit (QIAGEN) and finally resuspended in RNAse-free water.

**DNA Microarray Hybridization and Analysis**

Quality and integrity of the total RNA was controlled on an Agilent Technologies 2100 Bioanalyzer (Agilent Technologies; Waldbronn, Germany). 200 ng of total RNA were applied for Cy3-labelling reaction using the MessageAmp II-Bacteria Kit according to supplier´s recommendation (Ambion, Germany). As a result of IVT (*in vitro* transcription) reaction using aminoallyl-dUTP, antisense RNA were generated and subsequently coupled with fluorescent dye Cy3. Cy3-labeled aRNA was hybridized to Agilent´s 8x15k *Mycobacterium bovis* BCG Pasteur microarrays (GPL13749, GEO database, http://www.ncbi.nlm.nih.gov/geo/) for 16 h at 68 °C and scanned using the Agilent DNA Microarray Scanner. Expression values were calculated by the software package Feature Extraction 10.5.1.1 (Agilent Technologies; Waldbronn, Germany) using default values for GE1_107_Sep09 extraction protocol. Statistical analysis of the expression data was performed using the Gene Spring Software package (Agilent Technologies, Waldron, Germany). The complete data set has been submitted to the GEO database as accession number GSE49976.

**Quantitative Real-Time PCR (qPCR)**

The RNA amplification and microarray methodology used in this study was validated by qPCR analysis of the expression of selected *M. bovis* BCG genes. For each of two biological replicates, cDNA was generated from 1 μg total RNA isolated from RAW 264.7 macrophages re-infected with either ANC or SEL mycobacteria (see above) using a QuantiTect Reverse Transcription Kit (QIAGEN) according to the manufacturer’s instructions. The qPCR reaction mix consisted of the Fast SYBR Green Master Mix (Applied Biosystems), forward (-F) and reverse (-R) primers specific to the gene of interest (BCG_3107c virS –F: 5’-CGCTACCTCTACGTCCATTCG-3’, -R: 5’-GGACCGTGCGGTAGTTGATG-3’; BCG_1251 sigI –F: 5’-TTCCTCGACGATCTGCTCAA-3’, -R: 5’-GGGCAAAGGCGATGACATC-3’; BCG_1921c rpfC –F: 5’-GGCCACTTCCGGCGATAT-3’, -R: 5’-CCATGGCGGACTTGATGAG-3’; BCG_r17 16S rRNA –F: 5’-TCCCGGGCCTTGTACACA-3’, -R: 5’-CCACTGGCTTCGGGTGTTA-3’; BCG_3319c lat –F: 5’-GGGCATACCAGCAGTTGGAT-3’, -R: 5’-CGCATACCTGCGTCTTCTTG-3’) diluted to 10 μM in water and cDNA template diluted in water (1:100 for 16S rRNA, 1:50 for lat, 1:10 for sigI and virS, 1:5 for rpfC) in a total reaction volume of 20 μl. The qPCR reaction was performed in a MicroAmp Fast Optical 96-well Reaction Plate (Applied Biosystems, USA), sealed with MicroAmp Optical Adhesive Film (Applied Biosystems, USA) and run in the 7500 Fast Real-Time PCR System (Applied Biosciences, USA) with cycling conditions set to one cycle of 95 °C for 20 seconds followed by 40 cycles of 95 °C for three seconds and 60 °C for 30 seconds. Each experimental sample was analyzed in triplicate; the level of expression was determined relative to a standard curve generated using genomic DNA (diluted to 1000, 200, 100, 50, 25, 10 and 2 pg/mL). Data were analyzed using the 7500 Software v2.0.6 (Applied Biosystems, USA) and Microsoft Excel, the Ct values for each sample were calculated and referenced to the standard curve and averaged. Expression of each gene was normalised to the stably expressed 16S rRNA cDNA. The ratio of gene expression of the SEL to ANC strain was calculated for each gene and plotted as the fold change in gene expression with the standard deviation between the biological repeats displayed. Student’s t-tests were performed to verify that the differences in the fold change in gene expression between the selected strain and the ancestral strain were significant.

**Analysis of extracellular bacteria in LTI macrophages**

LTI macrophages were grown in 24 well plates for 4 days. The supernatant of the long-term infected cells were centrifuged at 150 x g and then frozen for CFU analysis. Cells were lysed with 1 mL of sterile water and also frozen. Supernatant and cell lysates were serially diluted in PBS-Tween 80 0.05 % and inoculated onto Middlebrook 7H10 complete agar medium. Colony-forming units were determined as the mean of three plates for the same condition after 4 weeks of incubation at 37 °C.

**Electron microscopy**

LTI macrophages were fixed in 4 % formaldehyde (FA in H_2_O), 5 % glutaraldehyde (GA in H_2_O) in 200 mM sodium cacodylate (pH 7.3, CaCo) for 10 minutes at room temperature (all reagents from Electron Microscopy Science, USA). Cells were then gently scraped, centrifuged and the pellet was incubated with 2 % FA, 2.5 % GA in 0.1 M CaCo (pH 7.3) for 1 h at room temperature for post-fixation. After several rinses in PBS (137 mM NaCl, 2.7 mM KCl, 10 mM Na_2_HPO_4_, 2 mM KH_2_PO_4_ in H_2_O, pH 7.2) with gentle centrifugation steps in between, the cells were post-fixed in 2 % OsO_4_ (Serva, Germany) in PBS for 1 h at room temperature and subsequently rinsed in PBS. Cells were dehydrated in a graded ethanol series and post-stained with saturated Uranyl acetate (Serva, Germany) during the 90 % ethanol step for 30 minutes at 37 °C. Subsequently, the residual water was removed with propylene oxide prior to embedding in epoxy resin (Epon 812, Serva).

**Nile Red staining**

Nile red stain 99 % and DMSO (Dimethyl Sulfoxide, Carl-Roth, Germany) were used for this method.  Nile red stain concentration was ≈0.5 µg/mL, and stain diluent was a 25 % (v/v) solution of DMSO with distilled water. LTI infected macrophages were incubated with the Nile Red solution 1:1000 in complete D-MEM medium and visualized by live cell imaging.

**Generation of BCG ANC Δ*pks1* strains**

Genomic regions of about 2 kb either upstream of *pks1* or downstream of *pks1* were obtained by PCR from BCG Pasteur genomic DNA by using the following pair of primers: F- 5’-GCGGCCGCGTGCACGCATGTATTTTGCT-3’, R- 5’-GCGGCCGCGTATAGCCCCTCAACATCC-3’, F- 5’-AAGCTTAGTATCGGTCCATGCCTAGC-3’, R- 5’-CTGCAGCTCGGAAGTCGTTGGAAGAG-3’, respectively (restriction sites are underlined). The amplified fragments were cloned into the p2NIL plasmid (12). The final delivery vector was generated by incorporation of the PacI cassette from pGOAL 17 into this last p2NIL recombinant vector. The vector generated was pre-treated with UV light (100 mJ × cm^−2^) to induce depurination and promote recombination (13). This UV-treated plasmid was used to electroporate the ancestral strain *M. bovis* BCG (ANC). The unmarked mutant was obtained using a two-step strategy described previously (12), and the mutant clones were identified by colony PCR, using primers that amplify a central region of the *pks1* gene (data not shown). The deletion of *pks1* from the mutant candidate strains was confirmed by southern blot analysis using the *pks1* gene as probe. The mutant strains resulting from allelic exchange were designated BCG ANC Δ*pks*1-1 and ANC BCG Δ*pks*1-2.

**Southern blotting**

Chromosomal DNA was prepared from the selected clones according to the method of van Soolingen et al. (14), digested with *Pst1* and then analyzed by Southern blotting (15) by using the region encompasses nucleotide 3002 to nucleotide 3248 of *pks1* as probe. The mutant strains resulting from allelic exchange were referred to as ANC Δ*pks1*-1 and ANC BCG Δ*pks1*-2.

**Re-infection of BMM with ANC, ANC BCG Δ*pks1*-1 and ANC BCG Δ*pks1*-2**

ANC, ANC Δ*pks1*-1 and ANC Δ*pks1*-2 mycobacteria were used to re-infect BMM (MOI: 2.5) in D-MEM supplemented with 10 % FCS, L-glutamine 2.5 mM, 5 % Horse serum (PAA, Austria) and 20 % L929 cells supernatant. After 1 h of uptake, cells were washed 3 times with PBS and fresh medium plus gentamicin 10 µg/mL (Sigma, Germany) was added. Cells were incubated for 1, 2, 3 or 6 days at 37 °C in 5 % CO_2_ atmosphere. Subsequently, cells were lysed in sterile water and serially diluted in PBS-Tween 80 0.05 %. Dilutions were plated in Middlebrook 7H10 complete agar medium. Colonies were counted after 3 to 4 weeks to determine CFU.

**Supplementary Figure Legends**

**Figure S1. Mycobacteria in LTI macrophages are mostly intracellular**

**A- B-** LTI cultures of macrophages were plated in presence or absence of 10 µg/mL gentamicin as indicated in the panel (A). Macrophages were lysed and plated at the indicated time points and CFU were calculated (B). No differences were observed when gentamicin was present to kill extracellular bacteria indicating that during this period of time, bacteria were within macrophages and protected from the antibiotic. Data represents the mean ± S.E.M of at least 3 independent experiments. **C-** Images showing Middlebrook 7H10 complete agar medium plates inoculated with the supernatant or lysed of LTI cells after centrifugation at 150 x g, in order to check the presence of extracellular bacteria in the LTI culture untreated with Gentamycin. **D-** Quantification of 15 replicates of the supernatant and lysed samples of LTI macrophages in the plates shown in (C) after 4 weeks of incubation. Data represents the mean ± S.E.M of at least 3 independent experiments. **E-** Quantitative analysis of the green fluorescence intensity associated to a single macrophage in the long-term infected culture during 48 h. LTI macrophages were plated and analyzed by live cell imaging for 2 days after plating. The plot is representative of one macrophage from 25 analyzed.

**Figure S2. Mycobacteria in the LTI macrophages are localized in autophagic compartments and accumulate neutral lipids in droplets**

**A- B-** Electron microscopy of LTI RAW 264.7 cells. Micrographs show the intracellular location of mycobacteria in late endocytic/autophagic compartments. Asterisks: selected mycobacteria. Scale bar: 1 μm (A-B), 200 nm (C-D). **C- D-** Mycobacterial lipid droplets in LTI macrophages. Micrographs show the dramatic accumulation of intracellular lipid droplets in mycobacteria from LTI cultures. Scale bar: 1 μm (C), 200 nm (D, inset. Asterisks: lipid droplets). **E-** LTI macrophages were stained with Nile Red and observed by confocal microscopy. Both, macrophages (main panel) and bacteria (E’, inset) showed positive staining for Nile red in droplets. Nu: nucleus. Scale bar: 10 μm. **F-** Quantitative analysis of Nile red fluorescence associated to mycobacteria in RI and LTI macrophages. Data represents the mean ± S.E.M of three independent experiments with at least 20 bacteria counted per condition, (**) p ≤ 0.01 from two-tailed Student’s t-test.

**Figure S3.** **Lipid dynamics of ANC vs. SEL mycobacteria *in vitro***

Isotopic labeling and incorporation of fatty acids associated to phospholipids (PL), glycolipids (GL) and neutral lipids (NL) in selected mycobacteria *in vitro* during 13 days of incorporation of ^13^C-acetate in 7H9 complete medium.

**Figure S4. Genotypic changes of SEL**

**A-** Diagram showing the strategy followed to map and compare the genomes of ANC and SEL mycobacteria. **B-** PGL production in ancestral and selected mycobacteria. Extraction of glycolipids and thin-layer chromatography (TLC) were performed in supernatants and pellets of ANC vs. SEL mycobacteria. Samples were loaded by duplicates. Image is representative of more than three independent experiments.

**Figure S5- Gene expression analysis during re-infection with ANC and SEL by microarray**

Heat map of the genes analyzed by microarray. Three independent samples were analyzed per duplicate in two different microarrays. The p-value cut-off was 0.05 and the fold-change cut-off ≥ 2.0. After analysis, 38 genes were differentially regulated (either up- or down- regulated), of them 14 were analyzed in duplicates or triplicates. Blue and red squares indicate genes down- or up-regulated in SEL respectively that were analyzed by qPCR.

**Figure S6**

**A-** Southern blot analysis of DNA samples digested with *PstI* and probed with nucleotide 3002 to nucleotide 3248 of *pks1*. Lane 1 and 3: ANC Δ*pks1*-1 and ANC BCG Δ*pks1*-2 clones. Lane 2: ANC strain. Arrow indicates the band corresponding to *pks1* and other bands correspond to homologous to *pks1* on BCG genome. **B-** PGL production in ANC, SEL, ANC Δ*pks1*-1 and ANC Δ*pks1*-2 clones. Extraction of glycolipids and thin-layer chromatography (TLC) were performed in pellets of the different strains of mycobacteria. Image is representative of more than three independent experiments. **C-** ANC, ANC Δ*pks1*-1 and ANC Δ*pks1*-2 clones were used to re-infect primary bone marrow macrophages (BMMs) as indicated above. After 1, 3 and 6 days of infection, the number of colonies was counted and CFU were calculated. Data represents the mean ± S.E.M of three independent experiments, (*) p ≤ 0.05, (**) p ≤ 0.01 and (***) p ≤ 0.001 from two-tailed Student’s t-test. **D-** Lung bacillary loads in BALB/c mice after intratracheal inoculation (1.25 x10^5^ CFU) of ancestral (ANC, green bars), selected (SEL, red bars) or Δ*pks1* BCG (ANC Δpks1-2, blue bars) bacteria The data represents the mean number of CFU ± SD in five mice of one representative experiment out of two independent experiments, (*) p ≤ 0.05 and (**) p ≤ 0.01 from two-tailed Student’s t-test.

**Table S1. Single nucleotide polymorphisms (SNP) pair wise comparison of ANC vs. SEL mycobacteria**

For the SNP analysis 18 single-nucleotide differences (coverage >20) could be found between the two strains and the reference, but no significant differences between them were found.

**Table S2. Deletion Insertion polymorphisms (DIP) comparison of ANC vs. SEL mycobacteria**

**Movies**

**Movie S1**- Expansion of the infection in LTI macrophages. LTI macrophages images were acquired throughout the indicated period (38.5 h). Each image was captured every 3.45 minutes. This video corresponds to Fig. 2A.

**Movie S2-** Propagation of the infection in LTI macrophages showing cell death, bacterial growth and efferocytosis. Images were acquired during 62 h and each image was acquired every 5 minutes. This video corresponds to Fig. 2D.

**Movie S3-** Cell division of LTI macrophages. Images from long-term infected macrophages were captured throughout the indicated period of time (37.5 h). The acquisition of each image was every 40 seconds. This video corresponds to Fig. 2E.

**Supplementary References**

1. Constant P, Perez E, Malaga W, Lanéelle MA, Saurel O, Daffé M, Guilhot C. (2002) Role of the pks15/1 gene in the biosynthesis of phenolglycolipids in the *Mycobacterium tuberculosis* complex. Evidence that all strains synthesize glycosylated p-hydroxybenzoic methyl esters and those strains devoid of phenolglycolipids harbor a frameshift mutation in the pks15/1 gene. *J Biol Chem* 11;277(41):38148-58.
2. Bligh, E.G. and Dyer, W.J. (1959) A rapid method for total lipid extraction and purification. *Can J Biochem Physiol* 37: 911-917.
3. Vancanneyt, M., Witt, S., Abraham, W.-R., Kersters, K. and Fredrickson, H.L. (1996) Fatty acid content in whole-cell hydrolysates and phospholipid fractions of pseudomonads: a taxonomic evaluation. *Syst Appl Microbiol* 19: 528-540.
4. Tillmann S., Strömpl C., Timmis K. N. and Abraham W.-R. (2005) Stable isotope probing reveals the dominant role of *Burkholderia* sp. in aerobic degradation of PCBs. *FEMS Microb Ecol*, 52:207-217.
5. Osterhout, G. J., Shull, V. H. & Dick, J. D. (1991). Identification of clinical isolates of Gram-negative nonfermentative bacteria by an automated cellular fatty acid identification system. *J Clin Microbiol* 29:1822–1830.
6. Craig, H. (1957) Isotopic standard for carbon and oxygen and correction factors for mass-spectrometric analysis of carbon dioxide. *Geochim Cosmochim Acta* 12:133-149.
7. Silfer, J., Engel, M., Macko, S. and Jumeau, E. (1991) Stable carbon isotope analysis of amino acid enantiomers by conventional isotope ratio mass spectrometry and combined gas chromatography/ isotope ratio mass spectrometry. *Anal Chem* 63:370-374.
8. Demmelmaier, H. and Schmidt, H.-L. (1993) Precise δ^13^C-determination in the range of natural abundance on amino acids from protein hydrolysates by gas chromatography - isotope ratio mass spectrometry. *Isotopes Environ Health Stud* 29:237-350.
9. Abrajano, T.A. jr., Murphy, D.E., Fang, J., Comet, P. and Brooks, J.M. (1994) ^13^C/^12^C ratios in individual fatty acids of marine mytilids with and without bacterial symbionts. *Org Geochem* 12:611-617.
10. Goodman, K.J. and Brenna, J.T. (1992) High sensitivity tracer detection using high-precision gas chromatography-combustion isotope ratio mass spectrometry and highly enriched [U-^13^C]-labeled precursors. *Anal Chem* 64:1088-1095.
11. Rohde KH, Abramovitch RB, Russell DG. (2007) *Mycobacterium tuberculosis* invasion of macrophages: linking bacterial gene expression to environmental cues. *Cell Host Microbe*. 2(5):352-64.
12. Parish T. and Stoker N. G. (2000) Use of flexible cassette method to generate a double unmarked Mycobacterium tuberculosis tlyA plcABC mutant by gene replacement,” Microbiology, 146 ( Pt 8):1969-75.
13. Kamath A. T., Fruth U., Brennan M. J. et al., (2005) New live mycobacterial vaccines: the Geneva consensus on essential steps towards clinical development. Vaccine, 31;23(29):3753-61.
14. van Soolingen D, Hermans PWM, de Haas PEW, Soll DR, van Embden JDA. Occurrence and stability of insertion sequences in Mycobacterium tuberculosis complex strains: evaluation of an insertion sequence dependent DNA polymorphism as a tool in the epidemiology of tuberculosis. J Clin Microbiol 1991;29:2578–86.
15. Southern, E.M. (1975): "Detection of specific sequences among DNA fragments separated by gel electrophoresis", J Mol Biol., 98:503-517. PMID 1195397.
